# A Bibliometric Analysis of Literature on the Impact of Rheumatoid Arthritis on Oral Health (1987-2024)

**DOI:** 10.7759/cureus.58891

**Published:** 2024-04-24

**Authors:** Namrata Dagli, Mainul Haque, Santosh Kumar

**Affiliations:** 1 Karnavati Scientific Research Center (KSRC), Karnavati School of Dentistry, Karnavati University, Gandhinagar, IND; 2 Pharmacology and Therapeutics, National Defence University of Malaysia, Kuala Lumpur, MYS; 3 Periodontology and Implantology, Karnavati School of Dentistry, Karnavati University, Gandhinagar, IND

**Keywords:** thematic analysis, overlay visualization, visualization, network visualization, dental health, periodontal health, oral health, rheumatoid arthritis, scientometric analysis, bibliometric review

## Abstract

This bibliometric analysis investigates the research landscape concerning the impact of rheumatoid arthritis (RA) on oral health through a comprehensive literature review. The study includes all English-language articles retrieved from the PubMed database, focusing on the relationship between RA and various aspects of oral health without any filter. The analysis of 261 publications revealed fluctuations in publication patterns from 1987 to 2024, with notable surges and declines in research activity. Collaborative networks among authors and institutions were identified, highlighting key contributors and prolific institutions such as Karolinska Institutet. The themes prevalent in the research included demographics, oral microbiota, biomarkers, treatment outcomes, and molecular mechanisms. Trend topic and thematic evolution analyses elucidated shifts in research focus from traditional concerns to emerging areas such as oral microbiology and immunological mechanisms. Key findings underscored the need for more clinical trials to comprehend the impact of RA on oral health, enhanced interdisciplinary collaboration, exploration of emerging areas, and longitudinal studies. This analysis provides valuable insights into the evolving research landscape, informing future investigations and interventions to improve oral health outcomes in individuals with RA.

## Introduction and background

Rheumatoid arthritis (RA) is a chronic autoimmune disorder characterized by inflammation of the joints, leading to pain, stiffness, and functional impairment [1]. Beyond its well-documented effects on joint health, emerging evidence suggests that RA may also significantly influence oral health and quality of life [2,3]. The bidirectional relationship between RA and oral health has garnered increasing attention recently, prompting numerous clinical trials to elucidate the complex interplay between these two domains [4].

Understanding the impact of RA on oral health is crucial, as both conditions share common underlying inflammatory pathways and may exacerbate each other's symptoms. While the epidemiological evidence connecting these two conditions remains limited, emerging scientific data suggests a potential link between certain bacterial pathogens found in periodontal disease, such as Porphyromonas gingivalis and Aggregatibacter actinomycetemcomitans, and the process of citrullination. This process may contribute to the formation of autoantibodies and impair the immune tolerance of individuals susceptible to RA [5]. Complications such as periodontal disease, temporomandibular joint disorders, and xerostomia have been reported in individuals with RA, underscoring the need for comprehensive management strategies that address both systemic and oral manifestations of the disease [6-8].

In this study, we aim to conduct a bibliometric analysis of the literature investigating the impact of RA on oral health. Bibliometric analysis is the quantitative examination of publication, citation, and collaboration patterns within scholarly literature to assess research impact and understand the dynamics of scientific knowledge dissemination. This analysis aims to delineate the existing research landscape and publication trends and identify key contributors, collaboration patterns, thematic focus, and emerging trends in this evolving field. This will provide valuable insights that inform future research endeavors and policy initiatives to improve the oral health outcomes of individuals with RA.

## Review

Material and methods

**Figure 1:**
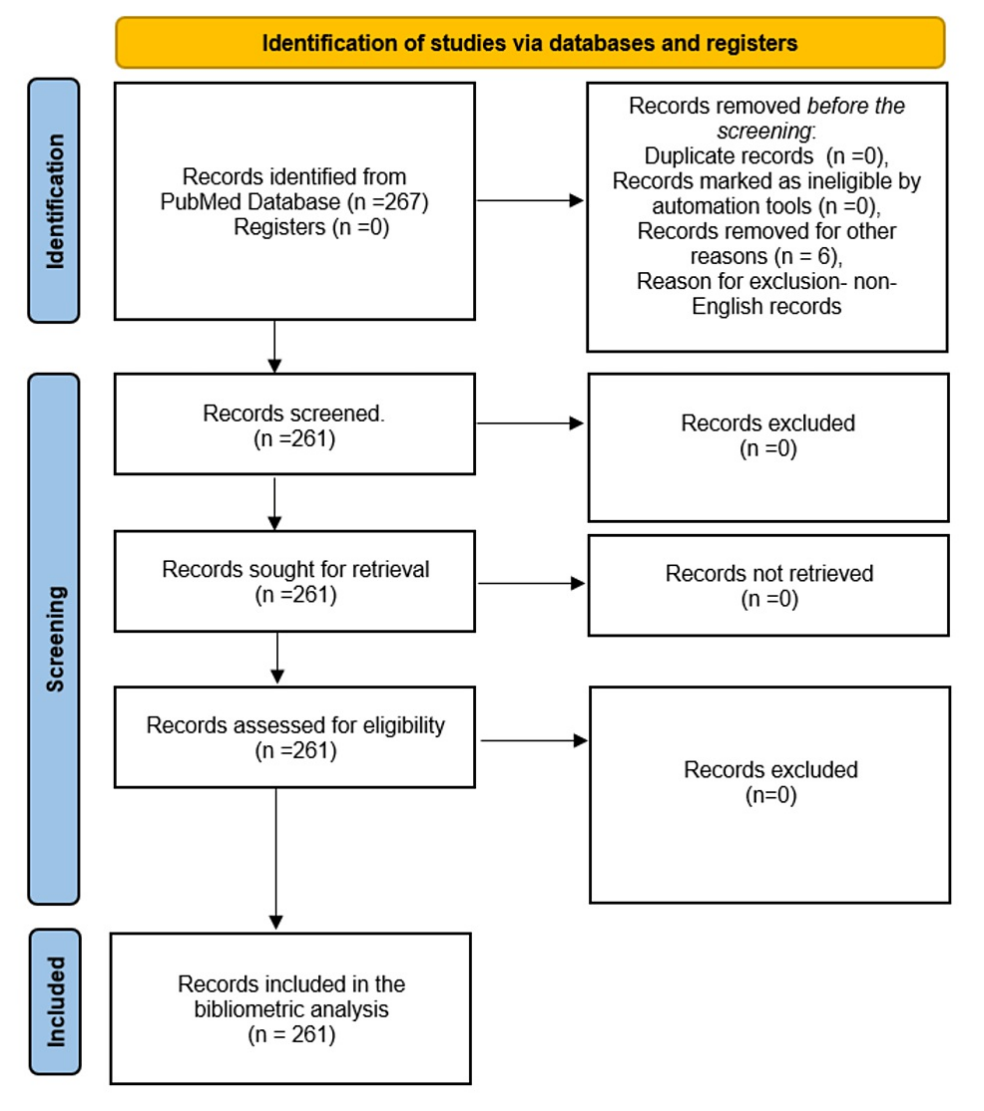
Flow chart of the study selection process. Image Credit: Namrata Dagli.

Using the PubMed database, an extensive search was conducted on April 9, 2024, targeting English-language articles focusing on the relationship between RA and various aspects of oral health. The following search string was used- ("rheumatoid arthritis" OR "rheumatoid arthritis-related") AND ("oral health" OR "periodontal health" OR "dental health"). Non-English articles were excluded using filters in the PubMed database. After the initial search, the extracted data were exported into a text file for further analysis. The process of selection of studies is depicted in the flow chart (Figure 1), generated according to the Preferred Reporting Items for Systematic Reviews and Meta-Analyses (PRISMA) guidelines [9]. Data analysis utilized software tools, including VOSviewer [10] for visualizing co-occurrence networks of keywords and author collaborations and Biblioshiny [11] for comprehensive bibliometric analysis and thematic analysis. Additionally, MS Excel and BioRender [12] were used to create graphical representations of the most relevant journals, affiliations, and key findings. The analysis involved examining publication trends, collaboration patterns among authors and countries, and keyword mapping to identify prominent themes and research trends. Ethical considerations were adhered to throughout the study process, ensuring respect for copyright and citation norms while utilizing data from existing literature. Limitations of the study included potential biases related to the availability of data within the PubMed database, language bias towards English articles, and variations in study methodologies across included studies. The findings of this bibliometric analysis were compiled into a comprehensive report, presenting graphical representations, statistical summaries, and interpretive insights to facilitate understanding and dissemination of the results to relevant stakeholders, contributing to the advancement of knowledge in this critical area of healthcare.

Results

Search Results

A total of 267 articles were initially identified in PubMed. Upon filtering for articles published in English, 261 articles remained. Among these, the distribution by type was as follows: there were eight case reports, seven clinical trials, one clinical trial protocol, four meta-analyses, 62 reviews, three observational studies, two editorials, and three comments. This breakdown underscores the diversity of literature available in PubMed, with reviews comprising the largest category, followed by case reports and clinical trials. Other types, such as meta-analyses, observational studies, editorials, and comments, represent smaller portions of the total corpus. The process of study selection is depicted in Figure 1.

Publishing Trends

**Figure 2:**
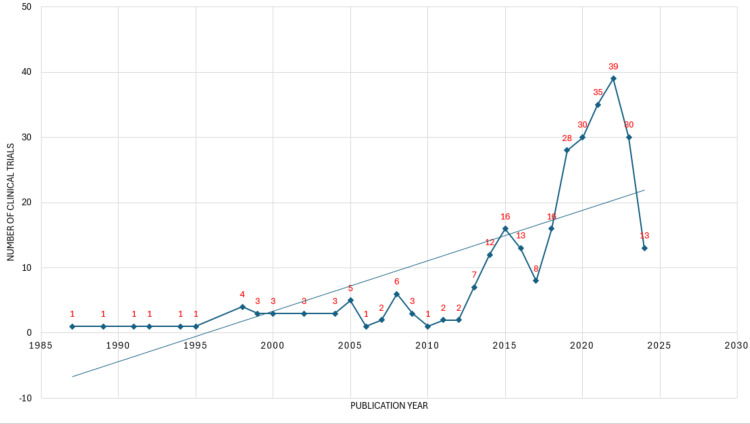
Annual scientific publications on the impact of rheumatoid arthritis on oral health. Image Credit: Namrata Dagli.

The publication pattern from 1987 to 2024 exhibits irregular fluctuations, characterized by periods of surges followed by declines, resulting in spikes in the curve. However, despite these fluctuations, the trend line suggests an overall increase since 1995. This indicates a general upward trajectory in the number of publications over time, albeit with fluctuations in intensity. Notably, the highest surge in publication occurs between 2018 and 2019, indicating a significant increase in research output during this period. Conversely, the highest decline is observed between 2022 and 2023, suggesting a sudden drop in publication activity during this time frame. Interestingly, the maximum number of papers was published in 2022, indicating a peak in research productivity. This could be attributed to factors such as increased funding, technological advancements, or emerging research trends that spurred a surge in publication activity. Between 1999 and 2004, the number of publications remains relatively constant. This period of stability suggests a plateau in research output, where the number of publications neither significantly increased nor decreased (Figure 2).

Most Relevant Authors

**Figure 3:**
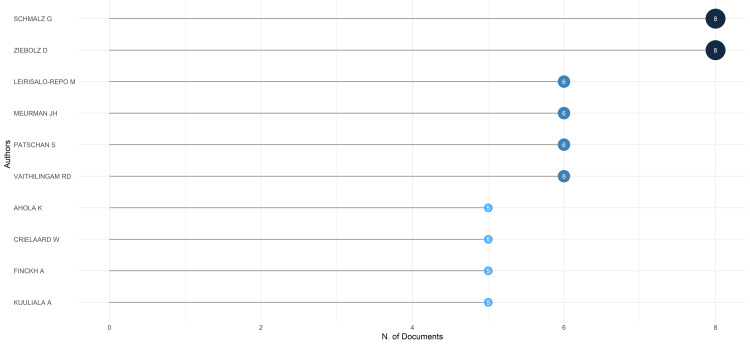
Most relevant authors based on the number of publications on the impact of rheumatoid arthritis on oral health. Image Credit: Namrata Dagli.

The authors Schmalz G and Ziebolz D stand out as the most prolific based on the number of publications, with eight publications each (Figure 3). The top 10 authors account for nearly 23% of all publications in the PubMed database. Interestingly, Schmalz G and Ziebolz D, the two leading authors, collectively contribute 26.67% of the publications authored by the top 10 most prolific authors.

Coauthorship Analysis of Authors

**Figure 4:**
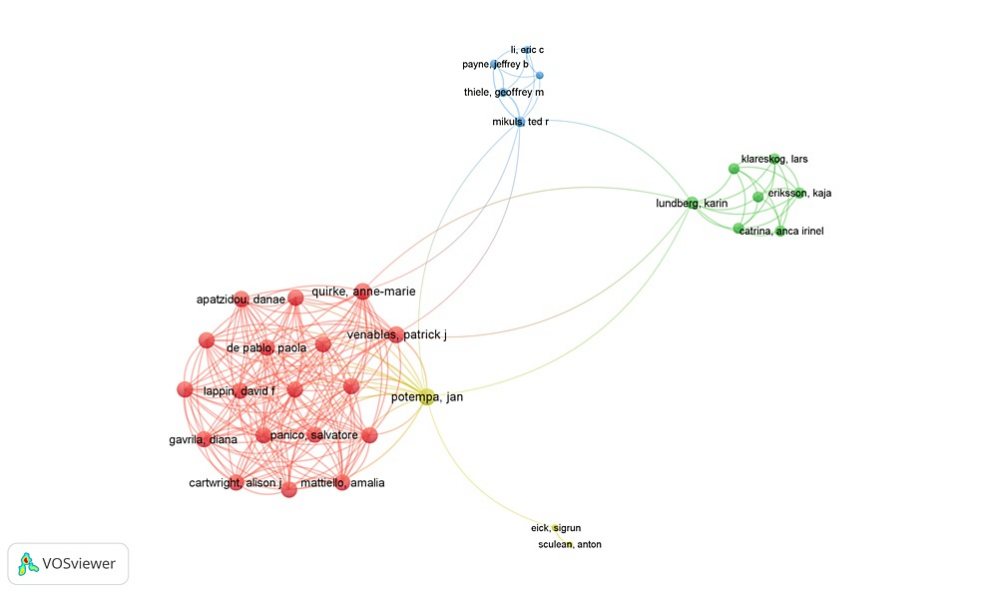
Network visualization of coauthorship analysis of authors. (Weight: Total link strength). Cluster 1 is red, Cluster 2 is green, Cluster 3 is blue, and Cluster 4 is yellow. Image Credit : Namrata Dagli.

The VOSviewer application identified 1371 authors, among whom 175 authors met the criteria of having a minimum of two publications listed in the PubMed database. Each author's total strength of coauthorship links was calculated using VOSviewer, where the total link strength (TLS) represents the overall strength of connections between authors based on their co-authorship relationships. All 175 authors with two publications were included in the analysis, and a network visualization was generated using VOSviewer. The top 5 authors with the highest link strength are represented in Table 1. Notably, author Tarcilia Aparecida Silva emerged with the highest total link strength value of 70 with five publications, indicating significant collaboration within the identified network. The most extensive set of connected authors included 33 authors, spread across four clusters, with 211 links and 413 total link strengths, as illustrated in Figure 4.

**Table 1:** The authors with the highest total link strengths value.

S. No.	Authors	Number of Publications	Total Link Strength
1.	Tarcilia Aparecida Silva	5	70
2.	Lucas Guimaraes Abreu	4	57
3.	Sicilia Rezende Oliveira	4	57
4.	Joice Dias Correa	4	55
5.	Jan Potempa	5	55

Most Relevant Affiliations

**Figure 5:**
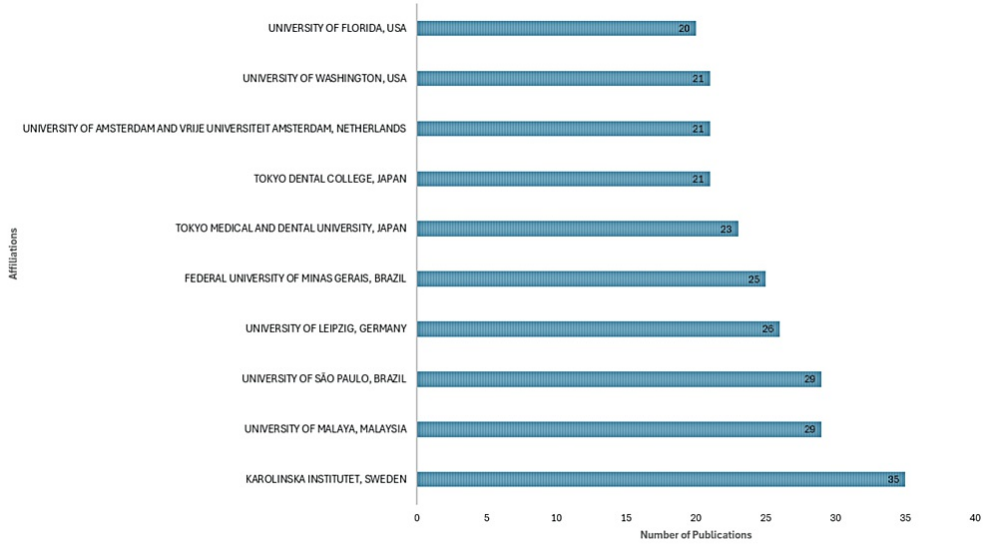
Most relevant affiliations based on the number of publications on the impact of rheumatoid arthritis on oral health. Image Credit: Namrata Dagli.

The research landscape in the field under consideration reveals a notable concentration of publications associated with specific academic institutions. Karolinska Institutet in Sweden is leading the pack, and it emerges as the most prolific contributor, reflecting its stature in medical research and education. The University of Malaya in Malaysia is closely behind, underscoring its significant involvement and impact on the subject area. Meanwhile, the University of Sao Paulo in Brazil also secures a strong position, showcasing its active participation in advancing scientific knowledge. However, what truly stands out is the contribution of the top ten institutes, as illustrated in Figure 5, which amounts to an impressive 95.8% of all publications in the PubMed database concerning this subject. This concentration highlights the influential role played by a select group of institutions in driving research and innovation within this domain.

Institution

**Figure 6:**
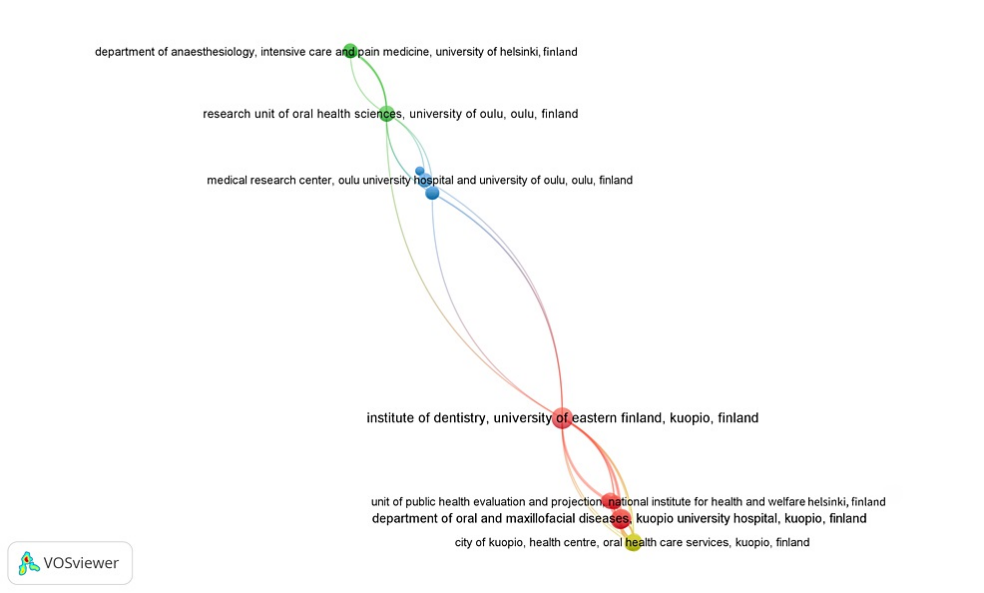
Network visualization of co-authorship analysis of institutions. (Weight: Total link strength). Cluster 1 is red, Cluster 2 is green, Cluster 3 is blue, and Cluster 4 is yellow. Image Credit : Namrata Dagli

The VOSviewer application identified a total of 901 institutions, among whom 34 institutions met the criteria of having a minimum of two publications listed in the PubMed database. Each institution's total strength of coauthorship links was calculated using VOSviewer, where the TLS represents the overall strength of connections between institutions based on their co-authorship relationships. The most extensive set of connected institutions included 24 institutions spread across four clusters, with 77 links and 86 total link strengths, as illustrated in Figure 6.

Co-occurrence of Keywords

**Figure 7:**
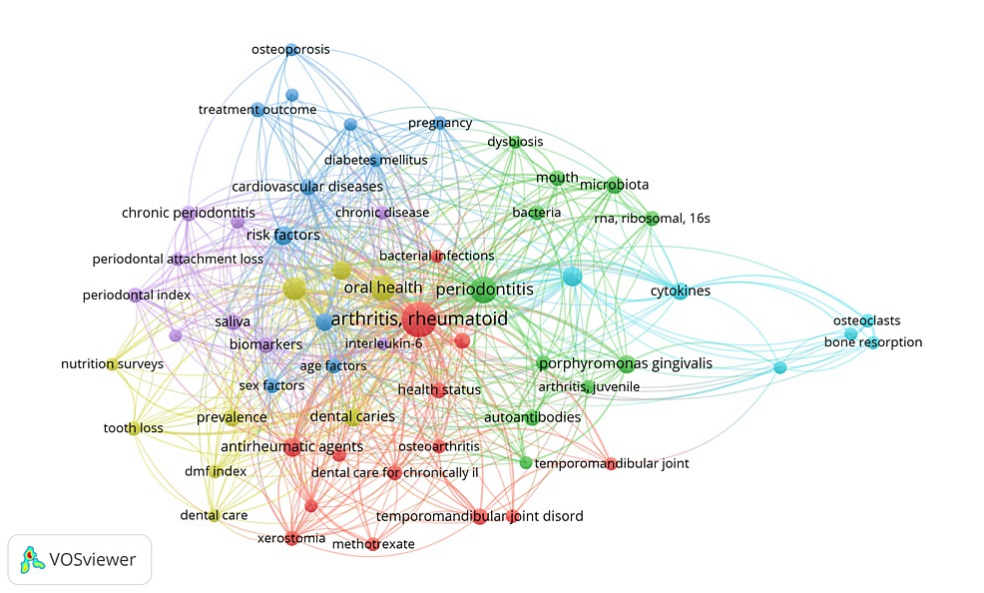
The network visualization of keyword co-occurrence analysis. Weight = Total link strength. Image Credit: Namrata Dagli.

VOSviewer identified a total of 677 MeSH keywords. When the threshold of minimum occurrence was set to 5, only 84 keywords met the criteria. For each of these keywords, VOSviewer calculated the total strength of the cooccurrence link. The 84 keywords were grouped under six clusters with 1524 links and 6173 TLS (Figure 7). The keywords in each cluster are represented in Table 2.

**Table 2:** Keywords in clusters identified in co-occurrence analysis of keywords.

Serial No.	Clusters	MeSH Keywords
1	Cluster 1 (20 items)	age factors, aged, aged 80 and over, dental care, dental care for chronically ill, dental caries, DMF index, logistic models, nutrition surveys, oral hygiene, patient education as a topic, periodontal diseases, prevalence, risk factors, sex factors, Sjogren's syndrome, smoking, tooth loss, xerostomia, young adult
2	Cluster 2 (17 items)	Bacteria, bacterial infections, cardiovascular diseases, chronic disease, diabetes mellitus, disease progression, dysbiosis, health status, humans, microbiota, mouth, mouth diseases, oral health, osteoporosis, pilot projects, pregnancy, rna ribosomal 16s
3	Cluster 3 (16 items)	Adult, antirheumatic agents, biomarkers, case-control studies, chronic periodontitis, Finland, follow-up studies, interleukin-6, methotrexate, middle-aged, periodontal attachment loss, periodontal index, periodontal pocket, prospective studies, saliva, severity of illness index
4	Cluster 4 (14 items)	Adolescent, anti-citrullinated protein antibodies, arthritis juvenile, autoantibodies, child, cross-sectional studies, female, male, osteoarthritis, peptides cyclic, periodontitis, Porphyromonas gingivalis, temporomandibular joint, temporomandibular joint disorders
5	Cluster 5 (9 items)	Animals, arthritis experimental, bone resorption, cell differentiation, cytokines, inflammation, mice, osteoclasts, pain
6	Cluster 6 (8 items)	arthritis rheumatoid, cohort studies, osteonecrosis, quality of life, research design, retrospective studies, surveys and questionnaires, treatment outcome

The keywords in cluster 1 suggest that the research likely involves examining age demographics, dental care practices for chronically ill individuals, specific oral health conditions like dental caries and periodontal diseases, statistical analysis using logistic models to identify prevalence and risk factors, consideration of sex differences, exploration of dietary habits and smoking, and the potential impact of autoimmune conditions like Sjogren's syndrome. RA and Sjögren's syndrome are distinct conditions. Still, they share some standard features due to their autoimmune nature, and individuals may sometimes have both conditions simultaneously or exhibit overlapping symptoms. Overall, it indicates a comprehensive approach to understanding how RA influences oral health and vice versa.

The keywords in cluster 2 indicate research focusing on the relationship between oral health, particularly the oral microbiota, and RA. The study aims to understand how oral bacteria and infections may impact overall health, including cardiovascular diseases, diabetes mellitus, and other chronic diseases. It also explores the progression of oral diseases and dysbiosis in the oral microbiota, considering their implications for health status. Osteoporosis and pregnancy are also relevant, suggesting investigations into potential connections with oral health. Using RNA ribosomal 16S sequencing indicates a microbiological approach to analyzing the oral microbiota concerning RA. The research appears to involve multidisciplinary studies, likely incorporating clinical, microbiological, and epidemiological methodologies.

The keywords in cluster 3 indicate research likely focused on the relationship between oral health, particularly chronic periodontitis, and RA. The study would involve adult subjects, possibly in Finland, and employ observational methods such as case-control and prospective studies. It would assess biomarkers, treatment outcomes (including antirheumatic agents like methotrexate), and disease severity through measures such as periodontal attachment loss and the severity of illness index. The study may also involve analyzing saliva samples to understand the link between immune responses (e.g., interleukin-6 levels) and the development or progression of both conditions.

The keywords in cluster 4 suggest that research likely involves studying different demographics, including adolescents, children, females, and males, to understand how these conditions manifest across various groups. Key aspects of the investigation may include autoimmune factors like anti-citrullinated protein antibodies and autoantibodies, as well as molecular mechanisms indicated by "peptides cyclic." Additionally, researchers might be interested in how RA and periodontitis impact the temporomandibular joint, which could involve cross-sectional studies examining data from individuals with both conditions simultaneously.

The research indicated by the keywords in cluster 5 likely explores how RA affects oral health using animal models. It focuses on bone resorption and inflammation, involving cell differentiation and cytokines. The aim is to understand arthritis-related pain and its impact on oral health.

The keywords in cluster 6 suggest a research focus on evaluating the impact of various RA treatments on quality of life. The research would likely involve longitudinal cohort studies tracking individuals over time, retrospective analyses of medical records, surveys/questionnaires to assess quality of life and oral health status, and evaluations of treatment outcomes. In addition, research would likely focus on evaluating the occurrence and severity of radionecrosis in individuals receiving radiation therapy for RA and its impact on overall treatment effectiveness and patient outcomes.

Analysis of Topic Trends 

**Figure 8:**
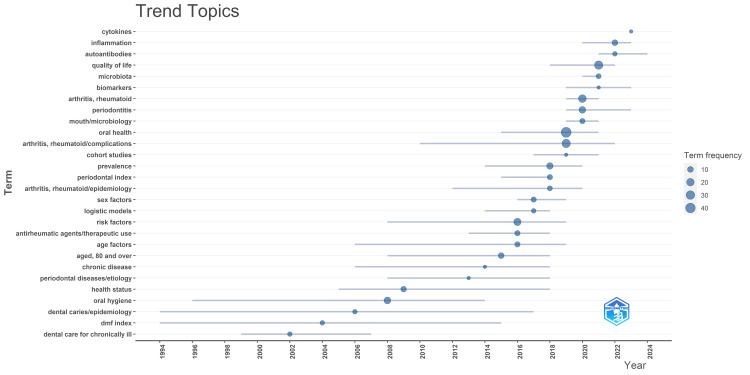
Analysis of topic trends in publications on the impact of rheumatoid arthritis on oral health. Image Credit: Namrata Dagli.

The purpose of topic trends analysis by Biblioshiny is to track and visualize the evolution of research topics over time. It helps researchers identify emerging areas, understand shifting dynamics, gain historical perspective, inform future research directions, and benchmark research productivity and impact. It is evident from the graph (Figure 8) that the research focus was initially on dental caries, periodontal diseases, oral hygiene, health status, and dental care for the chronically ill, particularly adults aged 80 and over.

After 2015, the focus shifted to the therapeutic use of antirheumatic agents and the epidemiology of RA. In addition, various risk factors, sex factors, and age factors were studied. After 2019, the research focus shifted to oral microbiology, microbiota, biomarkers, and quality of life, while sustained interest in periodontitis was noticed. Furthermore, the most recently used terms during the last two years are cytokines, inflammation, and autoantibodies.

Thematic Evolution 

**Figure 9:**
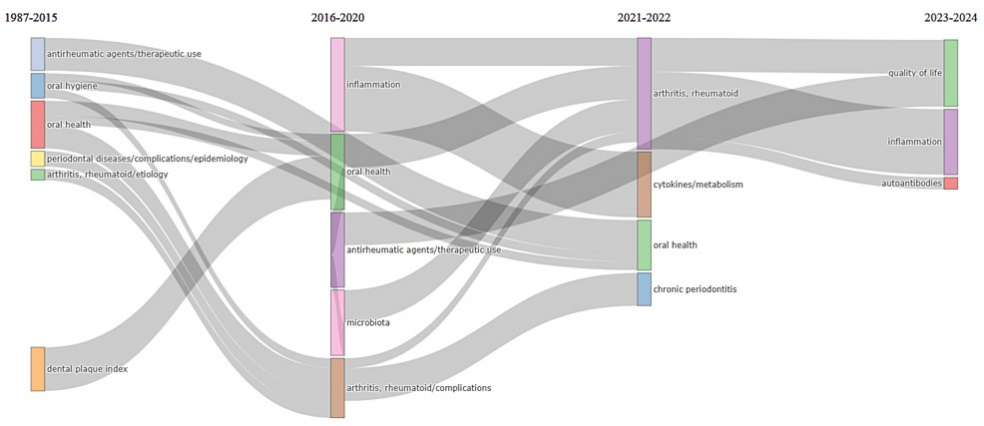
Analysis of thematic evolution (using Biblioshiny App) in publications on the impact of rheumatoid arthritis on oral health. Image Credit: Namrata Dagli.

The thematic evolution analysis depicted in Figure 9 visually represents how specific themes or concepts within a field of research have evolved. In this case, the analysis focuses on the evolution of themes related to oral health and associated topics. The graph suggests that certain themes, such as "oral health" and "oral hygiene," have decreased in importance over time. This could indicate a shift in research focus away from these traditional areas, perhaps due to research saturation or a perceived decline in relevance.

On the other hand, themes like "inflammation," "quality of life," and "autoantibodies" have been gaining more attention recently. This suggests growing interest in these areas within the research community, possibly driven by emerging discoveries and technological advancements. The thematic evolution analysis allows researchers to track these changes in research focus over time, providing valuable insights into the shifting dynamics of the field. By identifying which themes are gaining or losing importance, researchers can adapt their research priorities, anticipate emerging trends, and contribute to advancing knowledge within the field.

Thematic Map

**Figure 10:**
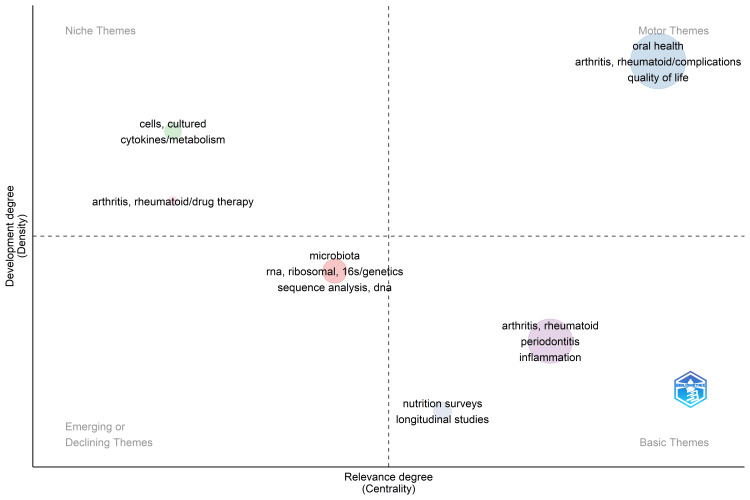
Thematic Map of keywords used in publications on the impact of rheumatoid arthritis on oral health. Field- keywords, Minimum frequency per thousand documents- 16. Image Credit: Namrata Dagli

Figure 10 presents a thematic map that categorizes themes into four distinct groups based on their development and relevance to the subject, likely RA and oral health. Within these categories, specific themes are identified to illustrate the landscape of research focus. Firstly, "Niche Themes" are highlighted, representing developed themes with limited relevance to the subject matter. Examples include "cultured cells," "cytokine metabolism," and "drug therapy of rheumatoid arthritis." These themes may have undergone substantial research and development but are deemed less pertinent to the core subject. Next, "Basic Themes," signifying relevant themes but not as firmly established, are periodontal inflammation, nutritional surveys, and longitudinal studies. These themes suggest that while relevant to the subject, they may not have received extensive attention or development compared to others. This indicated the need to address these related issues in the future. The "Motor Themes" category encompasses highly relevant and extensively developed themes within the subject domain. Themes such as "complications of rheumatoid arthritis," "oral health," and "quality of life" fall into this category, indicating their central importance and significant research attention. Lastly, the map highlights "Emerging and Declining Themes," which denote areas experiencing either increasing importance or diminishing relevance over time. This category includes "microbiota," "DNA sequence analysis," and "16s ribosomal RNA." These themes signify either burgeoning areas of interest or topics becoming less prominent within the research landscape. Overall, the thematic map offers a nuanced understanding of the various themes within the subject domain, providing researchers with valuable insights into the current state of research, areas of focus, and potential future directions for exploration.

Most Relevant Sources

**Figure 11:**
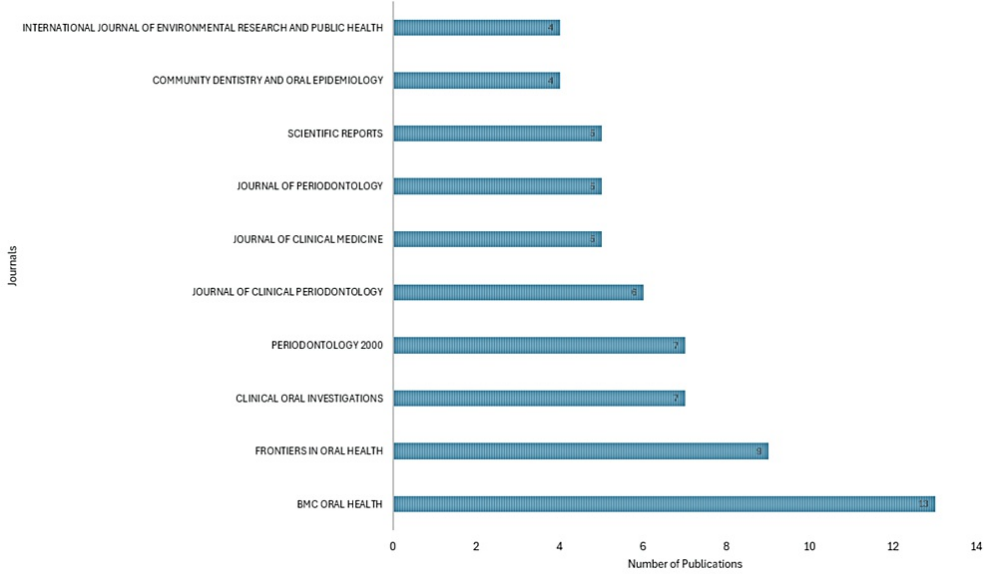
Most relevant journals based on the number of publications on the impact of rheumatoid arthritis on oral health. Image Credit: Namrata Dagli.

The analysis indicates that BMC Oral Health is the most prolific journal regarding the number of publications within the subject area, followed by Frontiers in Oral Health and Clinical Oral Investigation (Figure 11). These top three journals collectively account for a significant portion of the total publications in the field. Specifically, BMC Oral Health is the most prominent contributor, with its publications alone constituting 20% of the total output from all top ten journals combined. Furthermore, when considering the combined production of the top ten journals, they collectively contribute 30% of the total publications in the field. This underscores the importance of these top-tier journals in disseminating research findings and shaping the scholarly discourse within the subject area.

Most Relevant Countries 

**Figure 12:**
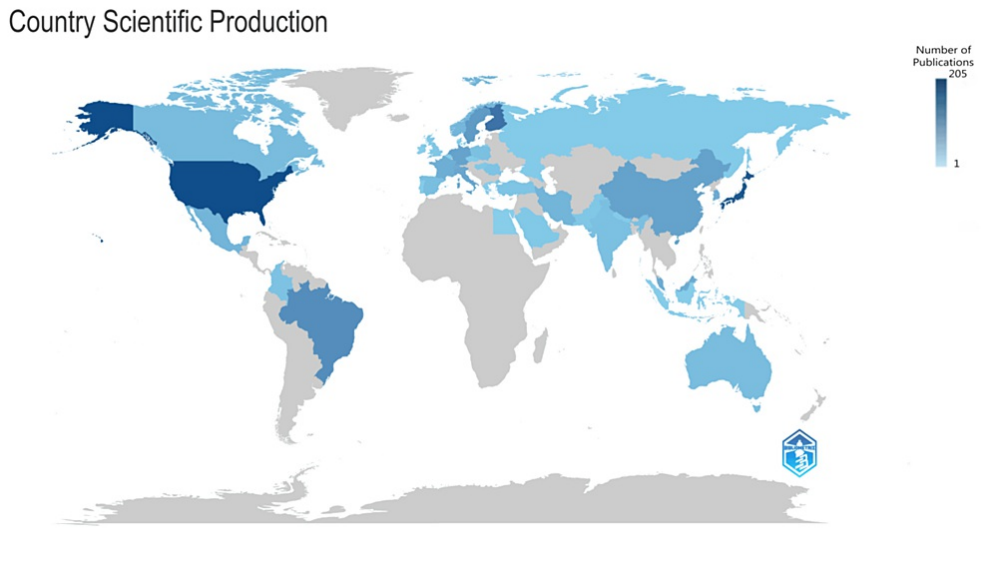
Scientific production of countries. Image Credit: Namrata Dagli.

The data reveals that the United States leads the pack with 205 publications, followed closely by Japan with 188 publications and Finland with 141 publications. Figure 12 visually represents the scientific production of countries, utilizing shades of blue to indicate the number of publications. The darker the shade of blue, the higher the number of publications attributed to that country. This color-coded visualization offers a clear and intuitive way to understand the distribution of scientific output across different nations. The dominance of the United States, Japan, and Finland in terms of publication output underscores their significant contributions to research within the subject domain. These countries likely possess robust research infrastructures, funding opportunities, and scientific expertise, enabling them to produce substantial scholarly work. The data sheds light on the scientific output of individual countries and provides insights into the global research landscape within the subject area.

**Figure 13:**
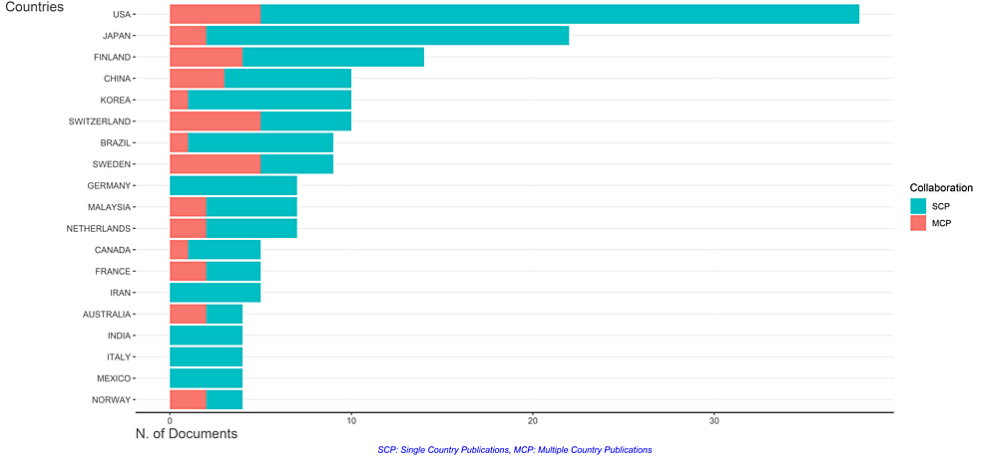
Most relevant countries based on the number of publications on the impact of rheumatoid arthritis on oral health. Image Credit: Namrata Dagli.

Figure 13 provides insight into the collaboration frequency among different countries in the field of research. The graph indicates that many publications are single-country efforts, meaning they involve researchers from within the same country working together on a project. On the other hand, the highest frequency of multiple-country publications is observed among certain countries. The United States, Switzerland, and Sweden are identified as leading contributors to collaborative research efforts, indicating a propensity for international collaboration among researchers from these nations. Following closely behind are Finland and China, suggesting a substantial engagement in collaborative research activities. Conversely, some countries exhibit minimal collaboration in their publications. Korea, Brazil, and Canada are noted for their low levels of collaboration, indicating a tendency for researchers within these countries to work independently or with minimal international cooperation. Moreover, the data highlights several countries, including Germany, Iran, India, Italy, and Mexico, that did not show any evidence of collaborative efforts in the publications analyzed. This suggests a lack of international collaboration among researchers from these nations within the subject area. Overall, Figure 13 underscores the varying levels of collaboration among different countries in research endeavors. While some nations actively engage in collaborative efforts, fostering knowledge exchange and sharing expertise globally, others exhibit a more insular approach to research, with minimal participation in international collaborations.

Discussion

The analysis reveals irregular fluctuations in publication patterns from 1987 to 2024, with periods of surges followed by declines. The significant surge in publications between 2018 and 2019 suggests intense research activity, possibly driven by technological advancements or emerging research trends. Conversely, the decline observed between 2022 and 2023 could be attributed to factors such as shifts in funding priorities or external events impacting research productivity. Despite an overall increase in publications since 1995, the number of publications on the topic is considerably lower. Clinical trials are required to understand the impact of RA on oral health. Only eight clinical trials have been conducted over 37 years, of which 4 were randomized controlled trials, and 1 was study protocol. 

These clinical trials collectively shed light on the intricate relationship between periodontal health and RA while offering insights into strategies for improving patient outcomes. Posada-López et al. demonstrated the positive impact of periodontal treatment on RA patients' health-related quality of life, underscoring the significance of periodontal care in enhancing overall well-being. However, the generalizability is limited by its quasi-experimental design, which could introduce biases and confounding variables [13]. Serban et al., through qualitative analysis in the OPERA trial, highlighted patient experiences and attitudes towards oral health in the context of RA, emphasizing the importance of understanding patient perspectives for effective intervention design. However, the full diversity of experiences among RA patients might not be captured [14]. Meanwhile, the trial by Monsarrat et al. assessed periodontal treatment's clinical and biochemical effects on RA patients, revealing a positive trend in the psychological impact domain of oral health quality of life. However, the sample size and duration of the study were less [15]. The study by Äyräväinen et al. investigated salivary and serum biomarkers in RA patients, showcasing salivary biomarkers' potential as indicators of RA periodontal health. The study may be limited by its cross-sectional design, which precludes the establishment of causal relationships [16]. The randomized controlled trial by Prado et al. illustrated the effectiveness of a personalized RA education tool in improving knowledge of RA risk factors among unaffected first-degree relatives (FDRs), emphasizing the importance of targeted educational interventions in RA prevention [17]. Self-selection bias and limited long-term follow-up to assess sustained knowledge retention might exist. Sparks et al.'s PRE-RA Family Study aimed to determine the impact of personalized RA risk education on behavior change in FDRs of RA patients, addressing challenges specific to targeting a high-risk population for RA [18]. While the study is innovative in its approach, it may encounter difficulties in achieving behavior change among FDRs of RA patients, given the multifactorial nature of RA risk factors. Finally, the study by Ribeiro et al. evaluated the clinical effects of periodontal treatment on disease severity markers in RA patients, suggesting a potential positive impact on systemic inflammation [19]. The study may be limited by its small sample size and lack of a control group, which could affect the robustness of the findings. These findings collectively inform our understanding of the complex interplay between periodontal health and RA, highlighting the potential benefits of personalized education and periodontal treatment in improving outcomes for RA patients. While these studies provide valuable insights, their limitations underscore the need for further research with larger sample sizes, extended follow-up periods, and rigorous study designs to confirm and build upon their findings.

Furthermore, our analysis identifies a network of authors collaborating on publications related to oral health and RA. Notably, author Tarcilia Aparecida Silva emerges as a key collaborator with the highest total link strength, indicating significant collaboration within the network. Multiple clusters suggest diverse collaborations among authors from different institutions and disciplines, fostering interdisciplinary research in this area. Karolinska Institutet is the most prolific contributor, followed by the University of Malaya and Sao Paulo. The analysis of institutional collaborations highlights the interconnectedness among research institutions studying oral health and RA. Multiple clusters indicate collaboration among institutions across geographical regions or specialization areas, facilitating knowledge exchange and research partnerships.

The analysis identifies key themes and topics in research on oral health and RA. Clusters of keywords represent distinct areas of investigation, including demographics, oral microbiota, biomarkers, treatment outcomes, and molecular mechanisms. These clusters reflect the multidisciplinary nature of research in this field, incorporating clinical, epidemiological, microbiological, and molecular approaches to understanding the relationship between oral health and RA. The analysis of topic trends reveals a dynamic evolution in research topics on the impact of RA on oral health, transitioning from traditional oral health concerns to exploring therapeutic interventions, epidemiological patterns, and emerging areas such as oral microbiology and immunological mechanisms.

The thematic evolution analysis reveals how specific themes in oral health research have evolved. While traditional themes like "oral health" and "oral hygiene" have declined in importance, newer themes such as "inflammation," "quality of life," and "autoantibodies" are gaining prominence. This evolution is likely driven by advancements in technology and evolving research interests. While trend topic analysis focuses more on broad topic trends and popularity shifts, thematic evolution analysis delves deeper into the field's conceptual changes and intellectual trajectories. Despite these differences in focus, both analyses contribute to a comprehensive understanding of the evolution of research within a particular domain.

The thematic map categorizes research themes into distinct groups, each reflecting different types of research within the domain of RA and oral health. "Niche Themes" represent areas with substantial development but limited relevance to the core subject matter, potentially involving cellular/molecular investigations or drug development studies. Conversely, "Basic Themes" signify relevant yet less firmly established research areas, often focusing on understanding connections like nutrition and periodontal inflammation's link to RA through clinical or epidemiological investigations. In contrast, "Motor Themes" denote highly relevant and extensively developed research areas, such as exploring the complications of rheumatoid arthritis on oral health and quality of life, often involving interdisciplinary studies and patient-centered outcomes research. Lastly, "Emerging and Declining Themes" indicate areas experiencing increasing or diminishing importance over time, revealing shifts in research priorities, such as emerging interests in microbiome research [20,21] and declining emphasis on previously extensively studied topics.

Regarding publication output, BMC Oral Health is the most prolific journal, followed by Frontiers in Oral Health and Clinical Oral Investigation. These top-tier journals collectively contribute a significant portion of the total publications in the field, indicating their importance in disseminating research findings and shaping scholarly discourse. Regarding country contributions, the United States, Japan, and Finland emerge as leading contributors to research on the impact of RA on oral health. These countries exhibit substantial publication output, likely owing to their robust research infrastructure and scientific expertise. Furthermore, collaboration patterns among countries vary, with some nations engaging in extensive international collaborations while others exhibit minimal collaboration in their publications.

**Figure 14:**
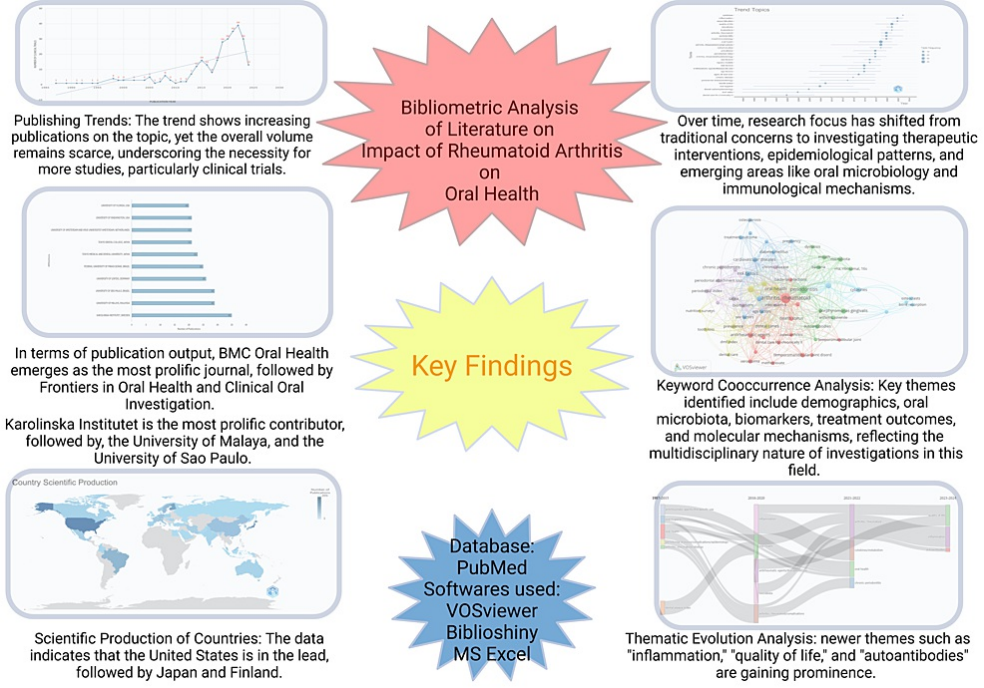
Key findings of the bibliometric analysis on the impact of rheumatoid arthritis on oral health. Notes: This figure has been drawn with the premium version of Biorender [12] (https://biorender.com/accessed on April 15th, 2024) with the agreement license number BD26P9I725. Image Credit: Namrata Dagli.

Our study offers a thorough insight into the research landscape of RA affecting oral health. Figure 14 encapsulates the key findings of our analysis. While previous bibliometric analyses have explored oral health and RA independently, none have delved into the intersection of these two conditions [22-28]. Notably, our study stands out as the first of its kind. Furthermore, we've strengthened our analysis by integrating qualitative theme analysis, enhancing the interpretation of our quantitative bibliometric findings. 

While this bibliometric analysis provides valuable insights into the research landscape surrounding the impact of RA on oral health, it is essential to acknowledge several limitations. Firstly, findings from a single database may not provide a comprehensive or accurate representation of the situation, emphasizing the need for confirmation from additional databases to validate the results. The analysis does not include individual paper analysis, which could offer more detailed insights into specific research methodologies, findings, and contributions. Furthermore, while the analysis identifies key collaborators, institutions, and research themes, it may overlook contributions from smaller research groups or non-academic stakeholders. Lastly, while trend topic analysis and thematic evolution analysis offer valuable insights into research trends and conceptual changes, they may not capture all nuances or interdisciplinary intersections within the field. Therefore, while the analysis provides useful insights into publication patterns, key collaborators, institutions, and research themes, caution should be exercised in interpreting the findings, and further research using multiple databases and individual paper analysis is warranted to gain a more nuanced understanding of the research landscape.

Future research on the impact of RA on oral health should prioritize conducting more clinical trials, particularly the microbiome-immune response dynamics in RA patients, long-term effects of periodontal treatment on disease activity and joint damage, influence of periodontal therapy on RA medication response, genetic and epigenetic factors contributing to RA and oral health interactions, effectiveness of patient education and behavior change interventions targeting oral health behaviors, interdisciplinary care models integrating dental and rheumatology professionals, and patient preferences in shared decision-making processes. In addition, it enhances collaborative efforts among researchers and institutions to foster interdisciplinary research and innovation, explore emerging areas such as oral microbiology and immunological mechanisms, focus on treatment outcomes and quality-of-life measures, employ longitudinal studies to track evolving research trends and encourage international collaboration to facilitate knowledge sharing and best practices. By addressing these priorities, researchers can deepen our understanding of the complex relationship between RA and oral health, leading to improved management strategies and better outcomes for affected individuals.

## Conclusions

The analysis of publications from 1987 to 2024 unveils dynamic fluctuations in research activity, marked by periods of surges and declines. Notably, the rise in publications between 2018 and 2019 suggests heightened research activity, while the subsequent decrease in publications from 2022 to 2023. Despite an overall increase in publications since 1995, the quantity remains very low, emphasizing the need for more robust research efforts, particularly clinical trials, to comprehend the impact of RA on oral health. The analysis further uncovers a collaborative network of authors and institutions, with notable contributions from key collaborators like Tarcilia Aparecida Silva and prolific institutions such as Karolinska Institutet. Institutional collaborations underscore the interconnectedness among research entities, fostering interdisciplinary partnerships and knowledge exchange. Key themes identified in the research include demographics, oral microbiota, biomarkers, treatment outcomes, and molecular mechanisms, reflecting the multidisciplinary nature of investigations in this field. Both trend topic and thematic evolution analyses contribute to a comprehensive understanding of research trends and conceptual shifts within the domain. Over time, the evolution of research topics and themes highlights a transition from traditional concerns to exploring therapeutic interventions, epidemiological patterns, and emerging areas such as oral microbiology and immunological mechanisms. Lastly, the prominence of journals like BMC Oral Health and contributions from countries like the United States, Japan, and Finland underscore their pivotal role in shaping scholarly discourse and advancing knowledge in this field. Overall, these findings offer valuable insights into the evolving research landscape surrounding the impact of RA on oral health, paving the way for future investigations and interventions.
